# Evaluation of immune protection induced by DNA vaccines from *Haemaphysalis longicornis* paramyosin in rabbits

**DOI:** 10.1186/s13071-017-2262-x

**Published:** 2017-07-06

**Authors:** Tian-Tian Zhang, Jin-Cheng Zhang, Xue-Jiao Cui, Jing-Jing Zheng, Ru Li, Fang Wang, Jing-Ze Liu, Yong-Hong Hu

**Affiliations:** 1 Key Laboratory of Animal Physiology, Biochemistry and Molecular Biology of Hebei Province, College of Life Sciences, Hebei Normal University, Shijiazhuang, 050024 China; 2 Shijiazhuang Posts and Telecommunications Technical College, Shijiazhuang, 050021 China

**Keywords:** *Haemaphysalis longicornis*, Pmy, DNA vaccine, Immune protection

## Abstract

**Background:**

*Haemaphysalis longicornis* is a blood-sucking ectoparasite that can cause diseases by transmitting some pathogens to humans and animals. Paramyosin (Pmy) is an immunomodulatory protein, which plays an important role in immune reactions against parasites. In this study, we evaluated the immune protection elicited by recombinant plasmids encoding *H. longicornis* Pmy in rabbits.

**Results:**

Rabbits vaccinated with pcDNA3.1(+)-Pmy developed high level of IgG compared to control group, suggesting that humoral immune response was induced by vaccination. On the fourth day after fed on the rabbit, some female adults died and the mortality rate from pcDNA3.1(+)**-**Pmy group (27.31%) was significantly higher than that of the control group (*P <* 0.0001). Other female ticks were attached to the rabbits until detachment, and the average engorgement weight, oviposition of female adult from pcDNA3.1(+)**-**Pmy group were 109.61 ± 4.24 mg and 48.39 ± 4.06 mg, respectively, which correspondingly resulted in 36 and 39% reduction compared with that of the control group (*P <* 0.0001). In brief, vaccination with Pmy plasmid DNA provided an overall efficacy of 50% in immune protection of rabbits.

**Conclusions:**

This study suggested that Pmy DNA vaccine can induce effective humoral immune response and partially protected rabbit against *H. longicornis* infection.

## Background

*Haemaphysalis longicornis* is an obligate hematophagous ectoparasite widely distributed in China, New Zealand, Korea, Japan and Australia [1]. Evidence showed that *H. longicornis* could transmit pathogenic microorganisms, such as *Babesia gibsoni*, *Theileria* spp. and *Coxiella burnetti* to humans and animals and cause diseases [2, 3].

So far, control of ticks is mainly dependent on the application of acaricides. However, utilization of acaricides causes drug resistance in ticks, environmental contamination, and meat products with pesticide residues [4–6]. Vaccine has been considered as an efficient approach to control ticks [7, 8]. DNA vaccines have some advantages, including the ability to induce both cellular and humoral immunity, rapid evaluation of candidate antigen designs, and less side-effects than vaccines based on attenuated pathogens [9, 10]. The *Toxoplasma gondii* 14–3-3 protein was a potential vaccine candidate and mice immunized with pSAG1/14–3-3 induced a high level of IgG antibody response, suggesting that pSAG1/14–3-3 was a novel DNA vaccine candidate against toxoplasmosis [11]. Genetic vaccination by injections of a plasmid containing the gene encoding the *Plasmodium berghei* circumsporozoite protein may be best for protecting against malarial sporozoite infection when the route of parasite entry is via mosquito bite [12]. Although many attempts to develop DNA vaccines against parasitic infections have been reported, few relative tests have been done in ticks.

Paramyosin (Pmy) is a myofibrillar protein existing in invertebrates, and the homologous Pmy does not exist in vertebrates. Thus, Pmy is one of the most ideal candidate antigen for vaccine development [13]. Previously, we successfully cloned the Pmy gene from *H. longicornis* [14]. In this study, an eukaryotic expression plasmid pcDNA3.1(+)-Pmy was constructed and used to vaccinate the rabbits. The relative biological parameters of female ticks were examined to evaluate immune protection induced by Pmy gene against ticks infections.

## Methods

### Ticks and animals

*Haemaphysalis longicornis* were collected in Xiaowutai National Natural Reserve Area in China by flag dragging and reared on rabbits as described by Liu et al. [15]. After detachment, ticks were collected and incubated in cotton-plugged glass tubes filled with folded filter paper in an incubator with 75 ± 5% relative humidity (RH) and 8/16 h of light-dark cycle (L/D) at 26 ± 1 °C.

New Zealand white rabbits weighing 2 kg were purchased from Hebei Medical University Laboratory Animal Centre (Shijiazhuang, China). Rabbits were maintained in a room with 50–55% RH at 25–27 °C and exposed to daylight. The protocol of all animal experiments was approved by the Animal Ethics Committee of Hebei Normal University.

### Recombinant plasmids

The total RNA of *H. longicornis* was extracted using AxyPrep™ Multisource Total RNA Miniprep Kit according to the manufacturer’s protocol (Axygen, San Jose, USA). cDNA was synthesized from 4 μg of total RNA by reverse transcription reaction using *EasyScript*® First-Strand cDNA Symthesis SuperMix (TransGen Biotech, Beijing, China). The open reading frame (ORF) of Pmy gene (GenBank Accession No. JQ517315) was obtained by PCR amplification using the following synthetic primers containing restriction sites underlined: forward primer: 5′-GAA TTC ATG TCT AGC AGG AGC AGC AAG T-3′ (*EcoR*I); reverse primer: 5′-GCG GCC GCC TAG AAG TTC TGG CTG GTC TCT T-3′ (*NotI*). The PCR product was doubled digested with enzymes and cloned into pcDNA3.1(+) with T4 ligase, the recombinant plasmid was named as pcDNA3.1(+)-Pmy. The plasmid was then transformed into *Escherichia coli* DH5α and sent to Invitrogen for sequencing.

### New Zealand white rabbit immunization and challenge

To evaluate the protective efficacy of the recombinant plasmid, New Zealand white rabbits were randomly divided into two groups (2/group). The experiment was repeated three times and 12 rabbits were used in total. Before vaccination, plasmids were diluted in buffer (10 mM Tris-HCl, pH 8.5) to a final concentration of 1 μg/μl. Control group and experimental group were injected intramuscularly with empty plasmid pcDNA3.1(+) (500 μg/each) or recombinant plasmid pcDNA3.1(+)-Pmy (500 μg/each) for three times with two weeks interval. Blood samples from rabbits were collected before the first vaccination, and the sera were obtained every seven days and stored at -20 °C for analysis. Ten days after the last injection, the rabbits of the two groups were challenged with unfed adults on the ears of rabbits (40 female ticks/ rabbit) and the ratio of female to male was 1:1. The stage of feeding blood was recorded every day until detachment of engorged female ticks, and the average engorgement weight, average egg mass weight, and hatchability, were also observed and recorded every day.

### Determination of antibodies by ELISA

Antibody levels in rabbit sera were determined by enzyme-linked immunosorbent assays (ELISA). Between each successive step, the wells were washed 3 times with PBS-Tween-20 (PBST). The 96-well microplates (Costar, New York, USA) were coated with 1 μg of tick antigens in 1 M carbonate buffer and incubated at 4 °C overnight. After blocking with 200 μl/well of blocking buffer (10% Bovine Serum Albumin in PBST) at 37 °C for 1 h, the plates were incubated with 100 μl/well of the rabbit sera which were serially double diluted from 1:200 to 1:204,800 in PBST for 45 min at 37 °C. Then 100 μl/well of HRP-conjugated goat anti-mouse IgG (Solarbio, Beijing, China) diluted 1:10,000 in PBST was added and incubated for 30 min at 37 °C. Finally, immune complexes were revealed by incubating with 100 μl/well of TMB Color liquid (Solarbio, Beijing, China) for 15 min at 37 °C in dark. The reaction was stopped with 50 μl/well of 1 M H_2_SO_4_, and the absorbance was measured at 450 nm with an ELISA reader (Molecular Devices, Sunnyvale, CA, USA). All samples were run in triplicate.

### Statistical analysis

Data in all groups were analyzed by using SPSS 12.0 software. Different biological parameters of female ticks were compared by Student’s *t*-test. The difference was considered statistically significant at *P* < 0.05. Vaccine efficacy was calculated as 100 × [1 – (NET × EWPF × H)], where NET, EWPF and H represent the fraction of the relevant tally in the experimental group relative to that in the control group of the total number of biting female ticks, total weight of eggs per female, and hatchability of eggs, respectively [16].

## Results

### Characterization of the antibody response in vaccinated rabbits

**Fig. 1:**
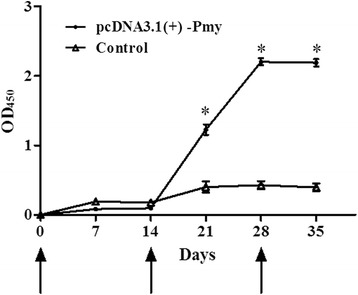
Antibody responses from immunized rabbits with DNA vaccine. Sera (2/group) were collected every seven days and determined by ELISA. The experiment was repeated three times. Results are shown as means ± SEM, and statistically significant differences are indicated by asterisks (*P < 0.05). The arrows indicate the days of the initial vaccination and the two boosters

The levels of IgG antibodies induced by plasmids in rabbits were detected every seven days by ELISA. The results showed that antibody levels in rabbits vaccinated with pcDNA3.1(+)-Pmy gradually increased after the second immunization (Fig. 1), and the IgG levels were significantly higher than those of the control group on the 7th day after the second immunization (*t*-test: *t*_(10)_ = 16.47, *P* < 0.0001). Furthermore, the IgG levels reached to the maximum value after the last immunization (Fig. 1).

### Rabbit tests for Pmy DNA vaccine against *H. longicornis*

**Fig. 2:**
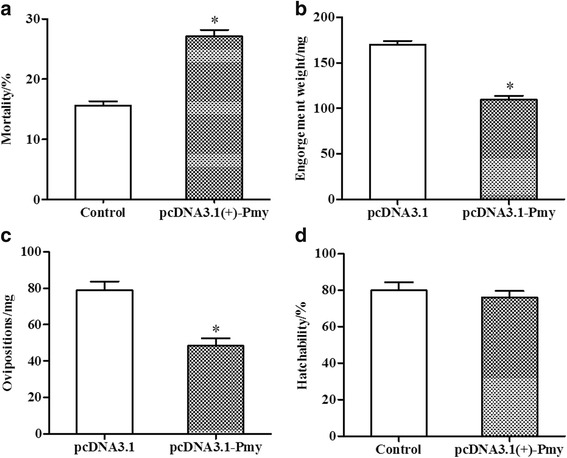
Different biological parameters of female ticks from rabbits tests. **a** Average mortality of female adults. **b** Average engorgement weight of female adults. **c** Average ovipositions of female adults. **d** Average hatchability. In total, 240 female adult ticks (*n* = 240) in Fig. 2a were used in each group, while biting ticks (*n* = 202 or 174) in Fig. 2b-d were used in control group and pcDNA3.1(+)**-**Pmy group, respectively. Results are shown as means ± SEM and statistically significant differences are indicated by asterisks (*P < 0.05)

**Fig. 3:**
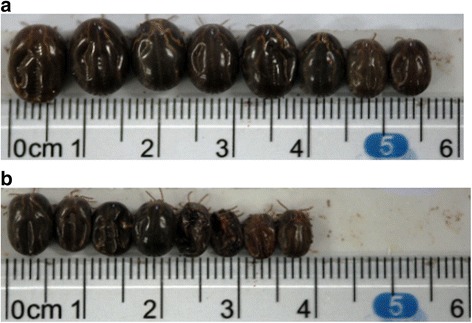
Comparison of tick size between two groups. **a** Control group (body width: 7.00 ± 0.38 mm), **b** Immunization group with pcDNA3.1(+)**-**Pmy (body width: 4.83 ± 0.23 mm). Female ticks were attached to immunized rabbits for blood-feeding until complete engorgement. Results are shown as means ± SEM

To evaluate the immune protection induced by the DNA vaccines of Pmy, all rabbits were challenged with ticks after immunization, and some biological parameters were checked daily. The results of the rabbit tests showed that some female adult ticks died on the fourth day after fed on the rabbit. It was possible that some ticks on the immunized rabbits ingested blood and that had an effect on the ticks feeding, which resulted in the death of ticks. The mortality of female adults from pcDNA3.1(+)**-**Pmy group was 27.31%, which was significantly higher than that of the control group (*t*-test: *t*_(10)_ = 9.09, *P* < 0.0001) (Fig. 2a, Table 1). Other female ticks were attached to the rabbits for blood-feeding until complete engorgement, and the average engorgement weight, oviposition from pcDNA3.1(+)**-**Pmy group were 109.61 ± 4.24 mg and 48.39 ± 4.06 mg, respectively (Table 1), which correspondingly resulted in 36 and 39% reduction compared with that of control group (*t*-test: *t*_(374)_ = 9.08, *P* < 0.0001; *t*-test: *t*_(374)_ = 4.72, *P* < 0.0001, respectively) in Fig. 2b and c. However, there was no significant difference in hatchability between the two groups (*t*-test: *t*_(343)_ = 0.09, *P* = 0.9279) (Fig. 2d). Analysis of the biological parameters indicated that the extent of the effect on female tick production resulted in an overall efficacy of 50% (Table 1). Furthermore, the mean width of female ticks fed on the pcDNA3.1(+)**-**Pmy vaccine group was 4.83 ± 0.23 mm, which was smaller than the mean width of those fed on the control group (7.00 ± 0.38 mm) (Fig. 3).

**Table 1:** Data from rabbits tests evaluating Pmy DNA for efficacy as anti-*H. longicornis* vaccine

Group	No. of animals	Total no. of ticks	Total no. of biting ticks	Mortality (%)	Feeding time (day)	Engorgement weight/female (mg)	Ovipositions/female (mg)	Hatchability (%)	NET	EWPF	H	Eff (%)
Control	6	240	202	15.63 ± 0.71	7.44 ± 0.08	170.20 ± 4.00	78.77 ± 4.96	79.93 ± 4.50	–	–	–	–
pcDNA3.1(+)-Pmy	6	240	174	27.31 ± 0.46^*^	7.50 ± 0.06	109.61 ± 4.24^*^	48.39 ± 4.06^*^	76.02 ± 3.58	0.86	0.61	0.95	50

NET = Reduction in tick numbers = Total number of biting ticks from the pcDNA3.1(+)-Pmy group / Total number of biting ticks from the control group

EWPF = Reduction in weight of eggs per female = The average eggs weight of female adult from the pcDNA3.1(+)-Pmy group/ The average eggs weight of female adult from the control group

H = Reduction in hatchability = Hatchability from pcDNA3.1(+)-Pmy group / Hatchability from control group

Eff = Overall efficacy compared to control = 100 [1-(NET × EWPF × H)]

^*^ *P* < 0.0001

## Discussion

It has been shown that Pmy is a promising vaccine candidate, which has been demonstrated in a variety of parasites, including *Schistosoma japonicum* [17], *Taenia saginata* [18] and *Trichinella spiralis* [19]. Studies on various parasites showed that Pmy DNA vaccine elicited higher levels of IgG, as well as cytokines IL-2, IL-4, IL-5, and IFN-γ. This demonstrated that Pmy plasmid DNA could induce both humoral and cellular immune responses [20, 21]. However, few studies have been reported in ticks.

In this study, rabbits were immunized with a novel Pmy DNA vaccine and the levels of IgG antibodies in rabbit sera were determined by ELISA. Our results indicated that antibody levels in the experimental group gradually increased two weeks after the first immunization (Fig. 1) and were significantly higher than those of the control group on the 7th day after the second immunization (*t*-test: *t*_(10)_ = 16.47, *P* < 0.0001). Li et al. [22] had verified that antibodies in mice immunized with Pmy recombinant plasmid from *Brugia malayi* were detectable two weeks after the first DNA injection and were significantly increased after the second injection. In addition, mice vaccinated with recombinant plasmid p14–3-3 developed higher levels of IgG on 2 weeks after the first immunization [11]. Our results also showed similar trend for antibody induction (Fig. 1). These results indicated that DNA vaccines induced a strong antibody response in rabbits or mice.

The protective efficacy of DNA vaccines against parasitic infections has been reported in other parasites besides ticks. Solís et al. [23] demonstrated that mice immunized with Pmy plasmid DNA of *Ta. crassiceps* caused 43 to 48% reductions of the parasite burden. Meanwhile, mice immunized with Pmy DNA vaccine from *T. spiralis* led to 44.8% of reduction in adult worm and 46.6% of reduction in muscle larvae after challenge with *T. spiralis* larvae [20]. Our results showed that vaccination of rabbit with Pmy DNA vaccine partially increased the mortality of female adult ticks (*t*-test: *t*_(10)_ = 9.09, *P* < 0.0001) (Fig. 2a), and this mechanism was not clear. In addition, the average engorgement weight and oviposition of female adult from pcDNA3.1(+)**-**Pmy group were significantly decreased compared with control group (*t*-test: *t*_(374)_ = 9.08, *P* < 0.0001; *t*-test: *t*_(374)_ = 4.72, *P* < 0.0001, respectively) (Fig. 2b, Fig. 2c), and the vaccine overall efficacy was 50% (Table 1). Moreover, female ticks from the pcDNA3.1(+)**-** Pmy vaccine group were smaller in size and reached to 31% reduction in body width compared with the control group (Fig. 3). This was similar to the effect of recombinant cyclin-dependent kinases (CDK) antigen on *Ixodes persulcatus* [24]. Thus, our results indicated that Pmy DNA vaccine was a potential vaccine candidate against ticks. To improve the vaccine efficacy, further studies with multi-gene vaccines should be conducted, and Pmy is one of the best choices.

## Conclusions

This study suggested that Pmy DNA vaccine elicited a specific humoral immune response and partially protected rabbits against *H. longicornis* infection. Furthermore, vaccination with Pmy plasmid DNA resulted in a significant reduction in engorgement weight, oviposition and size of female adult ticks in the vaccine group compared with the control group. This indicated that Pmy was a suitable candidate for the development of vaccine against ticks.

## Abbreviations

CDK: Cyclin-dependent kinases
ELISA: Enzyme-linked immunosorbent assays
EWPF: Reduction in weight of eggs per female
H: Reduction in hatchability
HRP: Horseradish peroxidase
IFN-γ: Interferon-γ
IgG: Immunoglobulin G
IL-2: Interleukin-2
IL-4: Interleukin-4
IL-5: Interleukin-5
L/D: Light-dark cycle
NET: Reduction in tick numbers
ORF: Open reading frame
PBST: Phosphate buffer saline-tween-20
Pmy: Paramyosin
RH: Relative humidity
TMB: Tetramethylbenzidine

## References

[1] Zheng H, Yu Z, Zhou L, Yang X, Liu J (2012). Seasonal abundance and activity of the hard tick *Haemaphysalis longicornis* (Acari: Ixodidae) in North China. Exp Appl Acarol..

[2] Yu Z, Wang H, Wang T, Sun W, Yang X, Liu J (2015). Tick-borne pathogens and the vector potential of ticks in China. Parasit Vectors.

[3] Lee DW, Chang KS, Min JK, Ahn YJ, Jo HC, Kim SI (2015). Acaricidal activity of commercialized insecticides against *Haemaphysalis longicornis* (Acari: Ixodidae) nymphs. J Asia Pac Entomol.

[4] Mendes EC, Mendes MC, Sato ME (2013). Diagnosis of amitraz resistance in Brazilian populations of *Rhipicephalus* (*Boophilus*) *microplus* (Acari: Ixodidae) with larval immersion test. Exp Appl Acarol..

[5] Zorloni A, Penzhorn BL, Eloff JN (2010). Extracts of *Calpurnia aurea* leaves from southern Ethiopia attract and immobilise or kill ticks. Vet Parasitol.

[6] Nong X, Tan YJ, Wang JH, Xie Y, Fang CL, Chen L (2013). Evaluation acaricidal efficacy of botanical extract from *Eupatorium adenophorum* against the hard tick *Haemaphysalis longicornis* (Acari: Ixodidae). Exp Parasitol.

[7] de la Fuente J, Kocan KM (2006). Strategies for development of vaccines for control of ixodid tick species. Parasite Immunol.

[8] Suarez M, Rubi J, Pérez D, Cordova V, Salazar Y, Vielma A (2016). High impact and effectiveness of Gavac™ vaccine in the national program for control of bovine ticks *Rhipicephalus microplus* in venezuela. Livest Sci..

[9] Myhr AI (2017). DNA vaccines: regulatory considerations and safety aspects. Curr Issues Mol Biol.

[10] Dowd KA, Ko SY, Morabito KM, Yang ES, Pelc RS, DeMaso CR (2016). Rapid development of a DNA vaccine for Zika virus. Science.

[11] Meng M, He S, Zhao G, Bai Y, Zhou H, Cong H (2012). Evaluation of protective immune responses induced by DNA vaccines encoding *Toxoplasma gondii* surface antigen 1 (SAG1) and 14-3-3 protein in BALB/c mice. Parasit Vectors.

[12] Weiss R, Leitner WW, Scheiblhofer S, Chen D, Bernhaupt A, Mostböck S (2000). Genetic vaccination against malaria infection by intradermal and epidermal injections of a plasmid containing the gene encoding the *Plasmodium berghei* circumsporozoite protein. Infect Immun.

[13] Zhang DM, Pan WQ, Qian L, Duke M, Shen LH, McManus DP (2006). Investigation of recombinant *Schistosoma japonicum* paramyosin fragments for immunogenicity and vaccine efficacy in mice. Parasite Immunol.

[14] Hu Y, Zhang J, Yang S, Wang H, Zeng H, Zhang T (2013). Screening and molecular cloning of a protective antigen from the midgut of *Haemaphysalis longicornis*. Korean J Parasitol.

[15] Liu J, Liu Z, Zhang Y, Yang X, Gao Z (2005). Biology of *Dermacentor silvarum* (Acari: Ixodidae) under laboratory conditions. Exp Appl Acarol..

[16] Guerrero FD, Andreotti R, Bendele KG, Cunha RC, Miller RJ, Yeater K, et al. *Rhipicephalus* (*Boophilus*) *microplus* aquaporin as an effective vaccine antigen to protect against cattle tick infestations. Parasit Vectors. 2014;7:475. 10.1186/s13071-014-0475-9 PMC4200143 25306139

[17] Zhou S, Liu S, Song G, Xu Y, Sun W (2000). Protective immunity induced by the full length cDNA encoding paramyosin of Chinese *Schistosoma japonicum*. Vaccine.

[18] Ferrer E, Moyano E, Benitez L, González LM, Bryce D, Foster-Cuevas M (2003). Cloning and characterization of *Taenia saginata* paramyosin cDNA. Parasitol Res.

[19] Chen X, Yang Y, Yang J, Zhang Z, Zhu X (2012). RNAi-mediated silencing of paramyosin expression in *Trichinella spiralis* results in impaired viability of the parasite. PLoS One.

[20] Wang L, Wang X, Bi K, Sun X, Yang J, Gu Y (2016). Oral vaccination with attenuated *Salmonella typhimurium*-delivered TsPmy DNA vaccine elicits protective immunity against *Trichinella spiralis* in BALB/c mice. PLoS Negl Trop Dis.

[21] Gu X, Xie Y, Wang S, Peng X, Lai S, Yang G (2014). Immune response induced by candidate *Sarcoptes scabiei* var. *cuniculi* DNA vaccine encoding paramyosin in mice. Exp Appl Acarol.

[22] Li BW, Zhang S, Curtis KC, Weil GJ (1999). Immune responses to *Brugia malayi* paramyosin in rodents after DNA vaccination. Vaccine.

[23] Solís CF, Ostoa-Saloma P, Lugo-Martínez VH, Johnston SA, Laclette JP (2005). Genetic vaccination against murine cysticercosis by using a plasmid vector carrying *Taenia solium* paramyosin. Infect Immun.

[24] Gomes H, Moraes J, Githaka N, Martins R, Isezaki M, Vaz Ida S (2015). Vaccination with cyclin-dependent kinase tick antigen confers protection against *Ixodes* infestation. Vet Parasitol.

